# Stingless Bee-Collected Pollen (Bee Bread): Chemical and Microbiology Properties and Health Benefits

**DOI:** 10.3390/molecules26040957

**Published:** 2021-02-11

**Authors:** Salma Malihah Mohammad, Nor-Khaizura Mahmud-Ab-Rashid, Norhasnida Zawawi

**Affiliations:** 1 Department of Food Science, Faculty of Food Science and Technology, Universiti Putra Malaysia, Serdang 43400, Selangor, Malaysia; salmamalihah94@gmail.com (S.M.M.); norkhaizura@upm.edu.my (N.-K.M.-A.-R.); 2 Natural Medicines and Products Research Laboratory, Universiti Putra Malaysia, Serdang 43400, Selangor, Malaysia

**Keywords:** stingless bee, bee bread, fermented pollen, phenolic compounds, microbes

## Abstract

Stingless bee-collected pollen (bee bread) is a mixture of bee pollen, bee salivary enzymes, and regurgitated honey, fermented by indigenous microbes during storage in the cerumen pot. Current literature data for bee bread is overshadowed by bee pollen, particularly of honeybee *Apis*. In regions such as South America, Australia, and Southeast Asia, information on stingless bee bee bread is mainly sought to promote the meliponiculture industry for socioeconomic development. This review aims to highlight the physicochemical properties and health benefits of bee bread from the stingless bee. In addition, it describes the current progress on identification of beneficial microbes associated with bee bread and its relation to the bee gut. This review provides the basis for promoting research on stingless bee bee bread, its nutrients, and microbes for application in the food and pharmaceutical industries.

## 1. Introduction

Bees have a unique social life. In a social bee community, the queen dominates the reproduction and has a morphology different from other bees. The colonies live for many years (perennial) [1]. Stingless bee is an example of social bees alongside honeybee and bumblebee. It contains more than 50 genera and 600 species worldwide [2]. Stingless bee is different from other bee species. As its name implies, stingless bee′s sting is significantly reduced [2] and is not an effective mechanism for nest protection, but it can still bite to protect its hive from threats.

Bee products are crucial for bee survival, and it is acquired for medicine, offering cosmetic, and everyday uses since 8000 BC [3]. Gradually, modern beekeeping industry emerged, whereby beekeepers collect these products for commercial purposes or personal uses. Stingless bees are widely distributed in countries such as South America, Africa, Southeast Asia, and Australia [1], prompting ventures of stingless bee beekeeping industry in these local regions for socioeconomic development and biodiversity conservation.

Bee bread is one the bee by-products made from pollen collected by the bee added with nectar and bee salivary enzymes before it undergoes lactic acid fermentation in beehives [4]. Bee bread is also referred to as fermented pollen, pot-pollen, stored pollen, or ambrosia [4,5]. Occasionally, among researchers, it is used interchangeably with bee pollen. In this article, the term bee bread is used and strictly referred to as stored pollen collected from inside of the beehives.

Consumer interest and demand for natural products have influenced in-depth research for bee bread nutritional properties. Bee bread is rich in carbohydrate, protein, and lipids and contain other micronutrients such as minerals, vitamins, phenolic compounds, and essential amino acids [6]. It possesses therapeutic values such as anti-inflammatory properties, antioxidant properties, antimicrobial properties, antitumor activity, and antihypertensive activity [6,7]. Bee bread nutritional values are varied as factors such as botanical origin, geographical location, climatic conditions, soil type, beekeeper activities, and bee species contribute towards its chemical composition [8,9].

Research on bee pollen from European honeybee (*Apis* spp.) has been continuously reported [4,6,10] overshadowing what bee bread can offer. One of the reasons is the difficulty in acquiring honeybee bee bread from the honeycomb [4]. This consequently influenced researchers′ preference to study on bee pollen. However, bee pollen is biochemically different to bee bread [11] as the latter is a product of fermentation. This makes research on bee bread essential to distinguish between these bee products for the development of meliponiculture industry. Apiarists could profit from bee bread production, which could sustain bee-keeping industry by not relying solely on honey as a source of income.

The nutrient richness of bee bread simultaneously promotes the growth of microbes. For the bee, microbes are essential to protect bee colonies against pathogens and provide nutrients for bee growth [12]. There is plenty of studies reported on isolation and identification of bee bread microbes with profound interest on lactic acid bacteria [13,14] because of its significant status as an industrially important group of bacteria. For instance, recent discoveries have identified bacteria from stingless bee bee bread as probiotic [15] and source of antimicrobial compounds and industrial enzymes [16] signifying the potential use of bee bread microbes in the food industry.

In this review paper, bee bread of stingless bee is introduced, highlighting its chemical composition and health benefits. In addition, microbes specifically associated with bee fermentation in relation to the bee gut are discussed.

## 2. Stingless Bee Bee Bread: From Production to Harvesting

The bee bread raw materials originate from the plant pollens. Pollens are fine granular substances produced by the plant anther containing male gametophyte (sperm cell), which are essential for plant reproduction. It is either distributed through the wind (“anemophilous”—wind-pollinated) or by an insect (“entomophilous”—insect-pollinated). The global production of pollens was estimated as 1.36 million kg per year with China, Australia, and Argentina as the biggest contributors [4].

**Figure 1:**
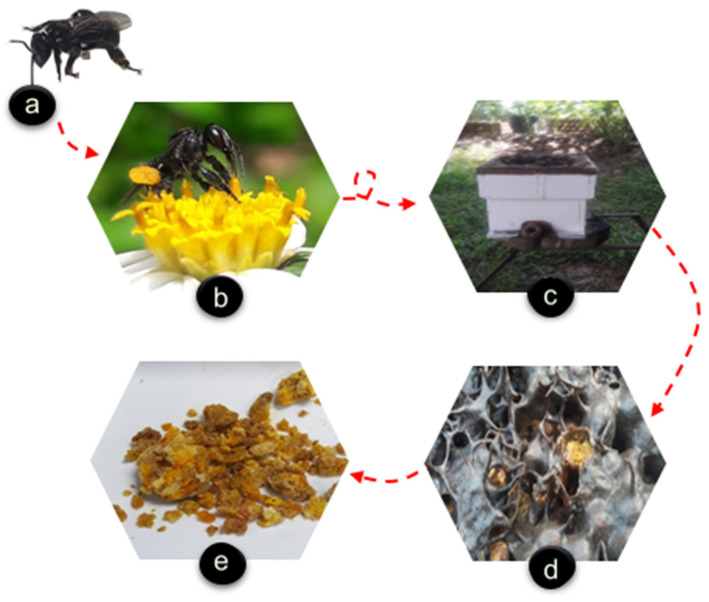
The production of bee bread from flower pollen. (**a**) Stingless bee, (**b**) stingless bee visits flower and collects pollen on its hind leg, (**c**) stingless bee returns to its hive, and (**d**) stores the pollen inside cerumen pots where lactic acid fermentation occurs to form bee bread. (**e**) Collected bee bread.

The process of stingless bee making bee bread begins from plant pollen, which is illustrated in Figure 1. Stingless bee can have an average flight range within a radius of 712 m [17] varying with bee species, bee′s body size, and food availability [18], [19]. Stingless bee such as *Heterotrigona itama* prefers foraging plants closest to their hive especially from white- and cream-coloured flowers [20,21,22] with nectar containing high sugar concentration [19].

During foraging, forager bees collect nectar and store it in their honey stomach while their bodies are covered in pollen dust. Pollen profile collected by different stingless bees species has been documented in Southeast Asia [21,22,23,24,25,26,27,28] and South America [29,30,31,32,33]. Stingless bees collect pollen from underutilised fruits, tree, and ornamental plants, shrubs, epiphytes, herbs, and lianas [21,31]. They have smaller body size compared to honeybees, which provides an advantage in collecting pollen from small flowers such as *Mimosa pudica* [22,26] and *Mimosa caesalpiniaefolia* [33].

As the bees collect pollens, they use their salivary enzymes (amylase and glucosidase) [34] and honey [35] to moisten, agglutinate, and pack the pollen into “pollen basket” on their hind legs [36]. The addition of these substances converts flower pollen into bee pollen. The forager bees transport the collected pollen (bee pollen) with nectar back to their hive. Some beekeepers collect the bee pollen, and this is usually observed in the case of honeybee *Apis*. Bee pollen is acquired using a pollen trap installed at the hive entrance, which strips the pollen from the bee′s leg by forcing the bees to crawl through a small tight hole [6].

**Figure 2:**
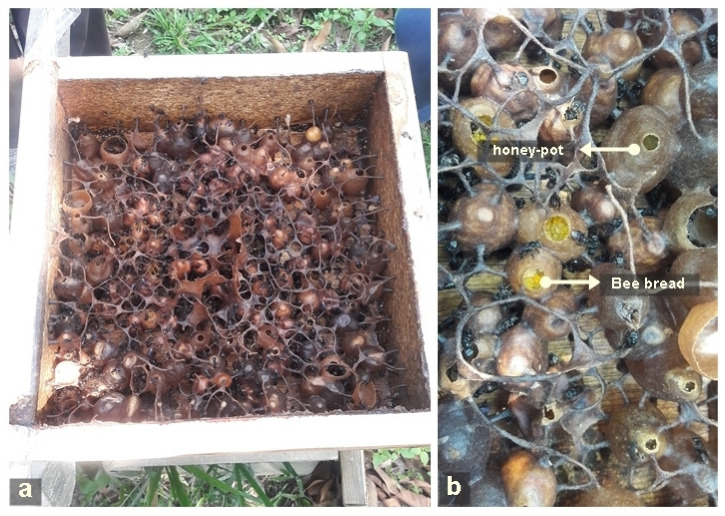
(**a**) Stingless bee colony and (**b**) honey and bee bread in separate cerumen pots.

If the bee pollen is not harvested, the worker bees pack the bee pollen inside a cerumen pot (for stingless bee) (Figure 2) or honeycomb cells (for honeybee) made from beeswax and resin. Once the pollen pots are full, the pots are sealed closed [37] before being consumed by larvae or young adult bees as a protein source [2]. During storage, lactic acid fermentation takes place to transform bee pollen into bee bread [38].

A single stingless bee colony could produce up to 6 kg of bee bread per year depending on the species. In Brazil, the maximum price for bee bread, locally known as “*sambura*” is 247 USD/kg [39]. In Malaysia, fresh wet bee bread could cost up to 95 USD/kg [22].

Because stingless bee′s bee bread is stored in cerumen pot, its acquisition is different from those of honeybee. Honeybee bee bread is acquired either through manual extraction [40] or usage of a specialised bee bread harvester for large scale production [41]. However, the current method to acquire stingless bee′s bee bread is using forceps, tweezers, or spatula. This imposes challenges especially towards mass production of bee bread for commercialisation [11].

### Biochemical Changes from Bee Pollen to Bee Bread

**Figure 3:**
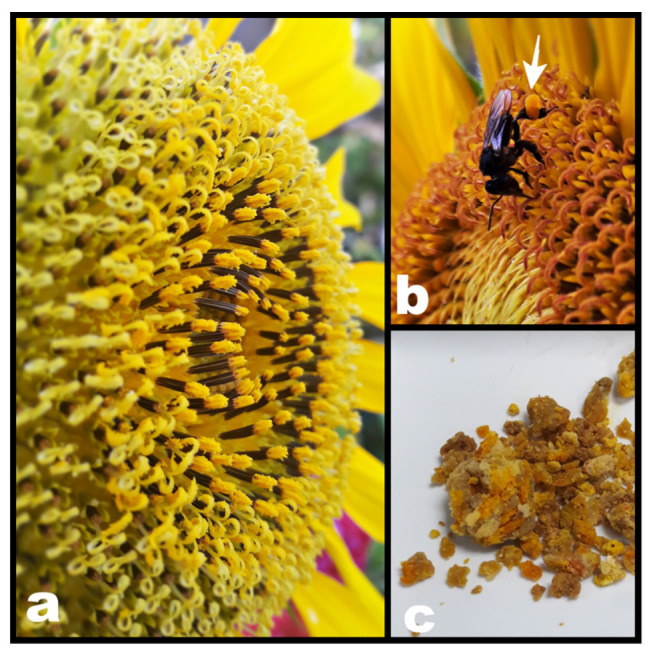
(**a**) Flower pollen, (**b**) bee pollen on the stingless bee (*Heterotrigona itama*) hind leg, and (**c**) fermented bee pollen (bee bread) collected from the stingless bee cerumen pot.

Flower pollen undergoes different development stages to become the end product known as bee bread. The biochemical profiles of flower pollen, bee pollen, and bee bread (Figure 3) are different as several biochemical changes take places at each stage [11,42]. Early studies revealed minor differences between bee bread and bee pollen. Bee bread lacks starch and has low ash content but has higher reducing sugar and fibre than bee pollen [43]. In the last decade, a few studies have attempted to compare the nutrient content of bee pollen and bee bread using more advanced technology. Although Anđelković et al. [42] found higher crude ash, protein, fewer minerals, and lower cellulose in bee bread than bee pollen, the botanical origins of the pollens which could influence the outcomes were not investigated.

Comparing the chemical composition of monofloral pollens is more enticing [6]. For example, investigating and comparing the monofloral fresh pollen, bee pollen, and bee bread of *Aloe greatheadii* var. *davyana* and *Helianthus anthuus* (sunflower) showed that bee bread has significantly higher water and carbohydrate content, but lower protein, lipid, and fatty acid content than its fresh pollen [44,45]. Flower pollen chemical profile was assumed to shift after collection because of the addition of bee salivary enzymes and honey [43,45]. However, according to researchers, these changes are considered minor [44,45].

Perhaps the noticeable change between bee pollen and bee bread is the pH. Bee bread is more acidic compared to bee pollen. Studies on pH changes of stingless bee pollen and bee bread are still lacking. However, when looking upon European bee pollen pH, it shows to be significantly reduced from pH 4.7 to 3.97 after transformation into bee bread [46]. Duarte et al. [47] also found high pH values (4.2–5.6) for *Melipona asilvai, M. quadrifasciata anthidioides, M. scutellaris, M. subnitida, Tetragona clavipes,* and *Plebeia* sp. bee pollen. The pH for stingless bee bee bread will be discussed in the later section.

Fermentation not only produces chemical changes in bee bread but also has been speculated to improve bee bread digestibility and bioavailability by degrading the outer pollen layer [48,49,50]. Nevertheless, Fernandes-da Silva [51] found no major difference in the nutritional value and digestibility between bee pollen and bee bread collected by *Scaptotrigona postica*. A recent study also showed bee bread digestibility equivalent to those of fresh pollen even after consumption and digestion by *Apis mellifera scutellata* [52]. In this sense, microbial fermentation was not able to ferment the highly resistant pollen walls to the fullest.

## 3. Physicochemical Properties

### 3.1. Physical Properties

A stingless bee can carry a pollen weight of 10.9 mg [53] and the pollen load decreases as the size of the bee increases [54]. The honeybee is larger than a stingless bee; therefore, it only carries an average of 7.9 mg pollen [55]. Pollen has diverse colours ranging from yellow, orange, brown, black, and purple which could indicate its botanical origin. For instance, bellflower (*Nesocodon mauritianum)* has purple pollen. Pollen colour tends to change into black once stored in hive due to oxidation [56]. Bee product’s colour is also associated with their phenolic content. The pollen colour is attributed to the presence of flavonoid [57], which relatively depends on the plant source.

Pollen wall has three different layers. The outermost layer is the pollenkitt followed by the outer wall (exine) and inner wall (intine). The exine consists of sporopollenin, while the intine is composed of cellulose and pectin [58]. The exine is the most difficult layer to be digested by bees and animals [59], and the structural integrity remains after storage of bee bread in the hive [51,52].

Bee bread contains accumulated pollen grains from various flowering plants. Although analysis of pollen colour could give an insight into its floral origin, a single pollen colour does not indicate monofloral source [60]. This is confirmed by Modro et al. [56] who suggested accurate pollen analysis for taxa identification by melissopalynology.

Melissopalynology is the microscopic study of pollen grains in bee products such as honey, pollen, propolis, and royal jelly [61]. Pollen grains are studied based on their physical features such as size, shape, aperture, and ornamentation [58]. The pollen grains sizes can be between 2.5 and 250 µm in diameter [10]. The surface of pollen has different structural properties such as clava, perforate, reticulate (mesh), granula, and echinate (spike-like) [58]. Each pollen is classified as either predominant, secondary dominant, or minor based on its frequency [62], which is subsequently important for product identification as either monofloral/unifloral or multifloral.

Pollen morphology is species-specific; therefore, it provides valuable information on the pollen’s botanical origin and plants preferentially visited by the bees [24,63]. This helps beekeepers plan and manage suitable landscape for bee foraging. Identifying plant origin also contributes to a better understanding of bee’s nutritional source, and bee products made from these plants, eventually, give commercial value to it [61].

The taste of bee bread is different across species. For example, *Tetragonula angustula*, *Ptilotrigona* and *Frieseomelitta doederlini*, and *F. varia* produce sweet bee bread, whereas *Melipona* and *Scaptotrigona* produce bitter bee bread [39,64]. To reduce the bee bread sourness, it is marketed mixed with honey or added in vitamins and juices [39].

### 3.2. Chemical Properties

Importantly, 250 different compounds comprise bee bread, and these are macro- and micronutrients, vitamins, amino acids, fatty acids, and phenolic compounds [65]. Bee bread has been dubbed as the “perfectly complete food” on various occasions because of its nutrient richness [66,67,68]. Bee pollens are consumed mainly as human food in Brazil and European countries such as Bulgaria, Poland, and Switzerland, and therefore, its physicochemical parameters and nutritional values have been standardised there [36]. Campos et al. [36] have proposed a quality criterion for honeybee *Apis mellifera* pollen to be considered as an international standard. However, unlike bee pollen, bee bread standard has yet to be articulated. Due to this setback, researchers opt to compare the research data of stingless bee bee bread to the international standard of bee pollen *Apis* spp. [22].

It is challenging to generalise bee bread nutritional values. Several factors including botanical and geographical origin, climatic condition, soil type, beekeepers’ activities, or storage treatments in commercial production [69,70] contribute to these challenges. The chemical properties of bee bread from stingless bee worldwide are summarised in Table 1.

#### 3.2.1. Water Content, Water Activity, and pH

Bee bread has shown to possess high moisture content. It is attributed to the pollen’s hygroscopic properties, which attracts water from the environment. Bee bread accumulates water from the surrounding and also through the addition of bee saliva and honey resulting in a sticky end product. Storage in closed pots prevents water loss from bee bread [71]. According to the bee pollen standard [36], the water content in pollen should not be more than 6–9/100 g.

Because pollen is hygroscopic, bee bread requires adequate drying parameters to achieve the desired moisture content. Different preservation methods are utilised in the beekeeping industry to preserve bee bread. Oven drying has been proven to be the best method to preserve bee bread over frozen and refrigeration method based on the reduction in moisture content and microbial load [72].

Bee bread has water activity between 0.60 and 0.92 (Table 1) within the range of A_w_ suitable for microbial colonisation, which is 0.60–1.00. The preservation method such as drying until A_w_ goes below this level and can help prolong the bee bread shelf life. High A_w_ of fresh bee bread provides a favourable environment for the growth of beneficial bacteria, pathogenic bacteria, fungi, or mould [15,73,74]. This creates concern over the risk of occurrence of mycotoxins, which has been reported in pollen [75]. Therefore, A_w_ parameter should be critically observed for quality control during storage.

As previously mentioned, bee bread is more acidic than bee pollen. Duarte et al.′s [47] study on seven species of stingless bee′s bee pollen in Brazil revealed that the pH values were in the range of 4.9–5.9. As shown in Table 1, pH values for stingless bee bee bread were reported to be in the range of 3.28–4.13. The lowest recorded pH was in one sample of bee bread of *Melipona scutellaris* (pH 3.28) [33]. The pH value dropped as the lactic acid content in bee bread becomes higher (3.06%–3.20%) than bee pollen (0.56%) [76] suggesting a lactic acid fermentation by bacteria and yeast [14] However, the exact mechanism is still poorly understood.

#### 3.2.2. Carbohydrates

Table 1 summarises the carbohydrate content in the stingless bee bee bread, which ranges from 10.85% to 59.94%. Carbohydrates mostly make up bee bread due to the addition of nectar during processing of pollen into bee bread. A significant increase in carbohydrates has been reported in two separate studies on monofloral bee bread [44,45]. The authors found higher carbohydrate content in *Aloe greatheadii* var. *davyana* and *Helianthus annuus* bee bread in comparison to its fresh pollen.

Most bee bread from Southeast Asia (SEA) is reported to have higher carbohydrate values than bee bread from other regions. Thailand′s *Tetragonula testaceitarsis* bee bread had the lowest value with 43.1% [80], while *Tetragonula biroi* Friese from the Philippines has the highest value with 59.94% [83] among SEA countries.

The sugar analysis comparison (Table 2) shows reducing sugars such as fructose, glucose, and sucrose were present in high amounts. Mannitol (sugar alcohol) was also found abundant in bee bread of *Tetragonula biroi* from the Philippines [83], *Meliponini subnitida* from Brazil [29,87], and *Trigona* spp. from Malaysia [88]. Other oligosaccharides such as sorbitol, cellobiose, isomaltose, maltose, raffinose, and stachyose were found in minute amount in *Tetragonula biroi* bee bread [83].

Bee incorporates nectar and its salivary enzymes during pollen agglutination [10]. Depending on bee species, honeybee *Apis mellifera* L. salivary (thoracic) gland secretes invertase, amylase, and glucosidase [89]. Stingless bee *Meliponini, Trigonini,* and *Scaptotrigona* spp. could digest pollen polysaccharides into simple sugar using enzymes α and ß-amylase and α-glucosidase [34]. Simple sugars are hypothesised to be fermented by bacteria during bee bread fermentation resulting in varying degrees of sugar fractions.

#### 3.2.3. Protein

Bee bread is rich in protein content, thus becoming the main protein source for bee development. The protein levels are varied across different geographical locations and between bee species (10.19–47.4%) (Table 1). Pollen with highest protein content was found to have originated from a highly nectariferous plant [90]. Although protein content decreases from fresh pollen to bee bread [44,45], whereas the amino acid concentration remains unchanged in fresh pollen, bee pollen, and bee bread [44,45].

Bee bread is claimed to contain all the essential amino acids–amino acids that cannot be synthesised by an organism [67,69,70], and it is indeed what was observed in the bee bread of stingless bee (Table 1). Belina-aldemita et al. [83] quantified the total free amino acids in *Tetragonula biroi* from the Philippines and found mean values of only 1.83 g/100 g bee bread. Among the essential amino acids, leucine, and phenylalanine were highly present in the studied bee bread (Table 3).

#### 3.2.4. Lipid

Lipid content of stingless bee bee bread is between 0.46% and 14.43% (Table 1). The outer pollen wall (pollenkitt) is made up of lipid [59,91]. Lipid is also present in the pollen grain at a different amount between the same and different species [92]. Bee bread lipid content is similar to bee pollen but significantly lower than flower pollen [45]. Addition of bee glandular enzymes into fresh pollen could alter the lipid composition available in bee pollen and bee bread.

Bee bread has diverse fatty acid profiles, which provides benefits to the bee nutrition and also for human health. According to Szczęsna [93], the most abundant fatty acids in bee pollen are linoleic (omega-6) followed by α-linolenic (omega-3) and palmitic acids. The fatty acid composition is not much different between bee pollen and bee bread [44]. Bee bread is rich in polyunsaturated fatty acids (PUFAs) such as omega-3, fatty acids which cannot be synthesised by our body [94]. Studies on fatty acid composition in stingless bee bee bread is still limited. For instance, bee bread of *Melipona scutellaris* contains 12 fatty acids (9 saturated and 3 unsaturated), and the most abundant saturated fatty acid was capric acid (1.89–5.66 g/100 g) and the common PUFAs were Omega-6 (α-linoleic acid) (0.50–1.63 g/100 g) and omega-3 (0.30–0.86 g/100 g) [33].

However, in another study, palmitic acid (1.65 g/100 g) was the prevalent fatty acid followed by omega-6 (1.52 g/100 g) in *Tetragonula laeviceps* bee bread. The ratio of polyunsaturated to saturated fatty acids was 1.59, higher than the ideal ratio of 1, which can slightly reduce HDL cholesterol level [80]. Similarly, palmitic acid was the dominant fatty acid among 15 fatty acids identified in *Melipona mandacaia* bee bread [85]. Omar et al. [88] analysed the volatile compounds in Malaysia *trigona* species using gas chromatography (GC-MS). Propanoic acid and palmitic acid were the most abundant saturated fatty acids in *Trigona apicalis* (4.04%) and *Trigona thoracica* (1.28%) bee bread, respectively. Meanwhile, minute amounts of omega-3 and omega-6 fatty acids were detected in range of (0.07–0.11%). In addition, analysis of volatile organic compounds (VOCs) found acetic acid as the sole organic acid in Australian *Tetragonula carbonaria* and *Tetragonula hockingsi* but absent in *Austroplebeia australis* [95].

#### 3.2.5. Phenolic Compounds

Phenolic compounds are secondary plant metabolites found widely in plants as a protective mechanism when encountering biotic or abiotic stress. They include phenolic acids, flavonoids, proanthocyanidins, and more. Intake of food containing phenolic compounds is sought to reduce the risk of gaining chronic diseases because of its antioxidant and anti-inflammatory properties [96]. According to findings of Urcan et al. [97], bee pollen and bee bread have similar phenolic profile. In contrast, Kaškonienė et al. [98] revealed that there is increase in flavonoid content (55–135%) after bee bread fermentation. Several studies have quantified the total phenolic content (TPC) and total flavonoid content (TFC) in stingless bee bee bread.

TPC and TFC content varies for different stingless bee species even from the same location [99]. The solvent of extraction also influences extraction efficiency. For instance, ethanolic extract and aqueous extract of *Heterotrigona itama* bee bread yielded different amounts of polyphenols. Ethanolic extracts were found to have higher TPC (21.32–22.54 mg GAE/g) and TFC (16.48–26.57 mg QE/g) than aqueous extract [78]. When comparing between ethanol and methanol, both solvents showed similar efficiency in extracting phenolic compounds from Australian *Austroplebeia* spp. and *Tetragonula* spp. bee bread. [100]. However, TPC and TFC of ethanolic extracts were slightly higher with 1281.2–2683.2 mg GAE/100 g pot-pollen and 282.9–698.0 mg QE/100 g pot-pollen, respectively. By far, ethanol has shown superior extraction for stingless bee bee bread compared to other solvents tested.

In Southern Venezuela, Vit et al. [84] determined the phenolic content and flavonoid contents in ethanolic extract of *Tetragonula angustula* bee bread. TPC ranged from 1053.1 to 2627.4 mg GAE/100 g pot-pollen, while the TFC was between 104.6 and 676.4 mg QE/100 g pot-pollen. In another study, Vit et al. [9] detected lower TPC and TFC from bee bread of seven stingless species of *Frieseomelitta* sp. and *Melipona* spp. with 1018.0–2085.0 mg GAE/100 g pot-pollen and 75.7–656.3 mg QE/100 g pot-pollen, respectively.

**Figure 4:**
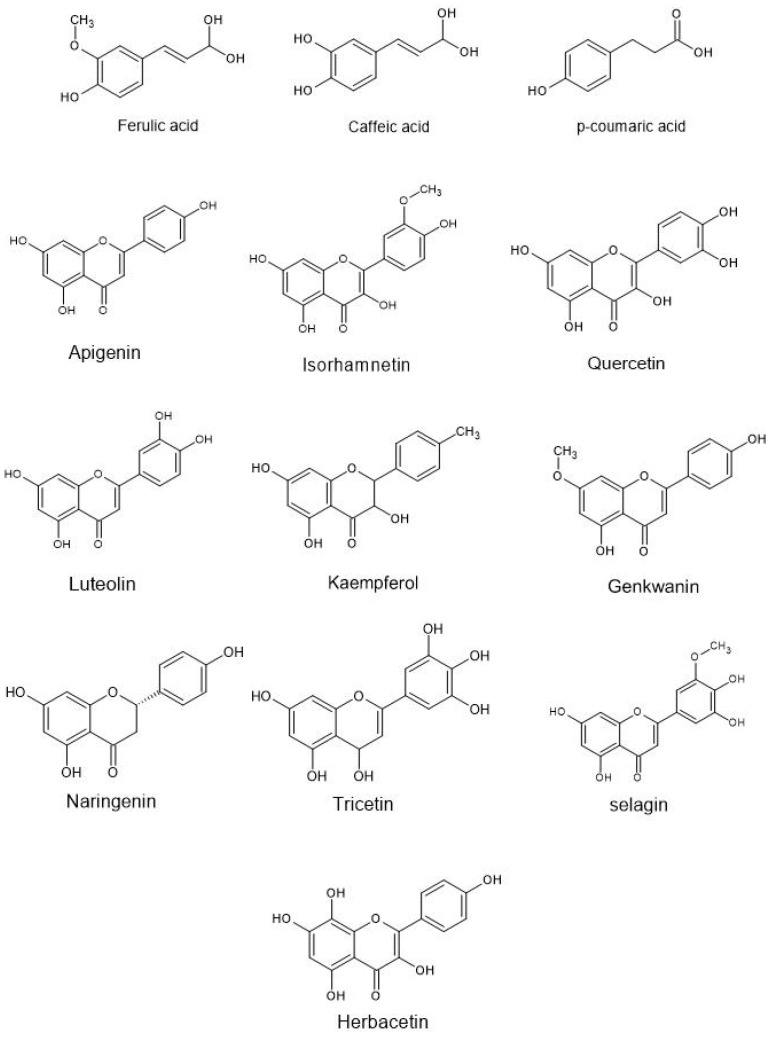
Chemical structures of some of the phenolic compounds found in stingless bee bee bread.

Table 4 summarises the bioactive compounds available in stingless bee bee bread. Figure 4 shows the chemical structures of the bioactive compounds. In general, kaempferol, isorhamnetin, and quercetin were the most frequently detected compounds in bee bread of stingless bee [9,29,78,101]. These molecules are also widely found in bee pollen of *Apis* spp. [4], fruits and vegetables, with great potential to reduce the risks of cancer [102,103].

#### 3.2.6. Other Micronutrients

Analysis of bee bread vitamin content is still scarce. *Heterotrigona itama* bee bread contains an average of 0.1108 mg vitamin C/g bee bread, which is considerably low based on the daily recommended intake [22]. Because vitamin C is unstable, some factors such as the method of preservation, drying parameter, and storage age and condition need to be optimised as they have shown to affect the vitamin content in pollen [60,108]. External factors, for instance, botanical origin, soil type, and climate are also suggested to influence vitamin C in pollen [109].

The most abundant mineral in stingless bee bee bread is potassium followed by phosphorus. From the studies conducted (Table 5), potassium was in the range of 2222.5–13,366.6 mg/kg. Similarly, it is also the most prevalent mineral in stingless bee honey [110]. Other minerals such as boron, rubidium, and strontium have been found in trace amounts in bee bread of *Tetragonula biroi* [83].

Apart from minerals, toxic metals such as arsenic, mercury, lead, and cadmium were detected in bee bread of *Heterotrigona itama* [22] and with a few *H. itama* samples exceeding the proposed limit for heavy metal [36]. Belina-aldemita et al. also detected these inorganic contaminants in *Tetragonula biroi* Friese bee bread but at a safe level [74]. Toxic metals accumulation in bee bread imposes high risk to human health but it can also become a good bioindicator of metal pollution in the environment [111] as a result of anthropogenic activities in nearby areas.

## 4. Health Benefits

There has been a tremendous report published on bee pollen as it is shown to possess therapeutic properties such as antioxidant, anti-inflammatory, antibacterial, anti-fungicidal, hepatoprotective, and anti-atherosclerotic activities [10]. In contrast, only a few publications have been reported on stingless bee bee bread’s biological properties, which are shown in Table 6.

### 4.1. Antimicrobial Properties

Bee bread extract contains phenolic compounds, which can be attributed to its antimicrobial properties. Therefore, its importance as an antimicrobial agent has to be acknowledged not only towards bacteria but also towards yeast and parasite. Carneiro et al. [112] demonstrated that bee bread extracts of stingless bee *M. compressipes manaosensis* inhibited *P. aeruginosa, M. smegmatis,* and *Candida albicans* efficiently and also mosquito larva *C. quinquefasciatus,* a vector for human parasitic worm *Wuchereria bancrofti*, in a concentration-dependent manner.

Akhir et al. [113] used ethanolic extract and hexanoic extract of *Heterotrigona itama* bee bread against *Bacillus cereus, Staphylococcus aureus, Escherichia coli,* and *Salmonella* sp. Gram-positive bacteria were more sensitive towards the bee bread extracts, whereas ethanolic extract demonstrated stronger antibacterial activity compared to hexane extract.

Meanwhile, Perez-Perez et al. [100] examined the antibacterial activities of ethanolic and methanolic extract from bee bread of *Austroplebeia australis, Tetragonula carbonaria*, and *Tetragonula hockingsi* in Australia. The extracts were effective against both Gram-positive (*Staphylococcus aureus*, *Bacillus subtilis*) and Gram-negative bacteria (*Enterobacter cloacae*, *Escherichia coli*, and *Pseudomonas aeruginosa*). Similarly, extraction using ethanol showed better results as shown by the lowest minimum inhibitory concentration recorded by ethanolic extracts of *Tetragonula hockingsi* bee bread.

Sulbarán-Mora et al. [114] investigated bee bread ethanolic extracts of *Frieseomelitta*, *Melipona*, and *Tetragonisca* spp. from Venezuela and found that they exhibited antibacterial effects against *Bacillus subtilis, Staphylococcus aureus, Enterobacter cloacae,* and *Pseudomonas aeruginosa* but not against *Escherichia coli.* The antibacterial activities were correlated to the phenolic content.

### 4.2. Antioxidant Properties

Phenolic compounds are one the most important natural antioxidants, which can be found in fruits, vegetables, tea, herbs, and essential oils. Antioxidant removes overproduced free radicals or reactive oxygen species (ROS), which cause molecular damages on DNA, protein, and lipid. Consequently, it has been linked with onset of diseases such as cancer and inflammation [115]. Stingless bee bee bread contains antioxidant compounds such as phenolic compounds and vitamin C [22]. Therefore, it has been explored for its ability to remove free radicals. Its antioxidant activity using in vitro study has been documented in Malaysia [79,99,113], Brazil [47,105,106], Venezuela [9,84], and Australia [100].

There are multiple methods to assess the antioxidant activity of a plant extract and 2,2,-di-phenyl-2-picryl-hydrazyl (DPPH) is the most common in vitro method applied due to its simplicity, cost effectiveness, and rapidity [116]. However, assessing using more than two in vitro models is more preferable because of the complexity of phytochemicals [117]. Therefore, bee bread antioxidant activity is assessed based on DPPH activity coupled with other antioxidant assays (Table 6).

Antioxidant activity is also affected by solvent extraction. For example, the antioxidant activities of ethanolic extract from *H. itama* bee bread were determined using DPPH and 2,2-azinobis-(3-ethylbenzothiazoline-6-sulphonic acid) (ABTS) [113]. The ethanolic extract showed higher DPPH and FRAP radical scavenging activity (93.60 ± 0.03% and 97.95 ± 0.01%. respectively) in comparison to hexanoic extract.

However, Perez-Perez et al. [100] found similar efficiency between ethanolic and methanolic extract of *Austroplebeia australis, Tetragonula carbonaria,* and *Tetragonula hockingsi* bee bread when using ABTS, Fenton type reaction, and hydroxyl radical, respectively. The antioxidant activities also varied according to bee species. Comparison between *trigona* species showed *Trigona thoracica* bee bread with the highest DPPH inhibition (IC_50_/0.86 mg/mL) followed by *Trigona apicalis* (IC_50_/1.05 mg/mL) and *Trigona itama* (IC_50_/3.24 mg/mL) [99].

Bee bread antioxidant ability corresponds to phenolic compounds [84,100,107,119]. Antioxidant activities also depended on the year of pollen collection, botanical origin, and storage time [120].

### 4.3. Antidiabetic Properties

Obesity is a public health concern. According to World Health Organisation [121], in 2016, more than 1.9 billion adults (18 years and above) were overweight with 650 million obese. A wide array of natural products has been assessed for their ability to combat obesity and that includes bee bread.

For example, *Heterotrigona itama* bee bread reduced Lee obesity index and levels of total cholesterol (TC), low-density lipoprotein (LDL), fatty acid synthase (FAS) activity, atherogenic index, oxidised-LDL (oxLDL), and malondialdehyde (MDA) and significantly increased aortic antioxidant enzymes activities (superoxide dismutase (SOD) and glutathione peroxidase (GPx)) in high-fat diet (HFD)-induced obese rats. En face aorta images showed smaller adipocytes sizes and absence of atherosclerotic plaque in obese rats supplemented with bee bread [104].

In another research, Eleazu et al. [118] studied bee bread effects on obesity-induced renal pathology. The outcomes showed 0.5 g/kg *H. itama* bee bread attenuated renal pathology caused by obesity by diminishing oxidative stress and downregulating the expressions of inflammatory markers and bax-mediated proapoptotic condition in the kidney of HFD obese rats.

### 4.4. Anti-Inflammatory Properties

Some studies also suggest bee bread extract’s ability to reduce inflammation. *Melipona fasciculata* and *Scaptotrigona affinis postica* ethanolic bee bread extracts were administered in induced-oedema mice model in two separate studies [105,106]. The anti-inflammatory responses were time and dose independent. After 5 h, the bee bread treatments were able to reduce the paw volume equivalent to the drug treatment of indomethacin (anti-inflammatory) and cyproheptadine (antihistamine). Further analysis identified the possible mechanism of phenolic compounds to inhibit the release of histamine and reduce prostaglandin synthesis [106].

### 4.5. Antinociceptive Properties

Bee bread extract has the ability to block pain detection through its antinociceptive activity. In vivo studies were conducted on pain-induced mice administered with bee bread ethanolic extract of two different species—*Melipona fasciculata* and *Scaptotrigona affinis postica*. Upon investigation, *Melipona fasciculata* bee bread extract (500 mg/kg) reduced the abdominal contort in mice suffering from acetic acid exposure better than indomethacin. Meanwhile, the formalin test showed bee bread extract efficiency similar to those of indomethacin in reducing biting/licking time in mice [106].

In a different study, Lopes et al. [105] investigated the antinociceptive activity using *Scaptotrigona affinis postica* ethanolic extract at lower dosage (250 mg/kg). The bee bread treatment showed similar effect to indomethacin in the acetic acid writhing test but significantly better than the drug (*p* < 0.005) in the formalin test. The presence of polyphenols and flavonoids was suggested to be responsible for these activities [105,106].

## 5. Microbiology Properties of Bee Bread

Bee pollen conversion into bee bread via lactic acid fermentation is linked with microbial action. Bee bread has been assumed to undergo microbial fermentation to increase its nutritional value [37,48]. This theory is termed as “Bee bread maturation” hypothesis [122], a notion that has been proposed since the mid-1990s but recently has been argued [123]. Through these studies, bee bread was found only to be preserved, and there was lack of evidence in nutrient conversion by microbes.

In this section, understanding the bee bread microbial ecology will include data of bee bread from other bee species such as the honeybee and solitary bee due to limited record on stingless bee bee bread microbes. Beneficial bacteria isolated from bee bread, such as LAB, *Bifidobacterium*, *Bacillus,* and yeasts from different bee bread species are summarised in Table 7.

Regardless, isolation and identification of microbes from bee bread have been widely reported. Yet, the study on their functional properties towards the bee ecosystem and most importantly, their potential for industrial application is still incomprehensive [37,124]. Some LAB from honeybee bee bread displayed antifungal activity towards foodborne pathogens *Aspergillus niger, Zygosaccharomyces rouxii,* and *Candida* sp. [73]. To date, Ngalimat et al. [16] identified *Bacillus* spp. from *Heterotrigona itama* bee bread with enzyme and antimicrobial-producing properties. In addition, Mohammad et al. [15] isolated lactic acid bacteria from the same stingless bee species with probiotic potential in vitro. This provides a basis to promote study for the application of beneficial microbes from bee bread in the food industry.

### 5.1. Lactic Acid Bacteria

Lactic acid bacteria (LAB) such as Lactobacillus, Enterococcus, Lactococcus, Leuconostoc, Pediococcus, Streptococcus, Carnobacterium, Aerococcus, Vagococcus, Oenococcus, Tetragenococcus, and Weisella [125] produce lactic acid as a major end product of fermentation. Undeniably, microorganisms such as LAB and yeast are associated with bee bread fermentation process, yet the details of the process are still vague with no conclusive explanation on the exact microorganisms responsible for this process [126]. An attempt to create artificial bee bread using Lactobacillus rhamnosus GG was made [98].

Current literature has conveyed extensive study conferring on microorganisms associated with honeybee bee bread or its ecosystem overall, particularly for *Apis mellifera* species. Only a few studies have discussed on microorganisms in the stingless bee [37]. The bacterial species found in bee bread across different bee species are diverse. *Lactobacillus* sp. particularly *Lb. kunkeei* is commonly found in bee bread [123,127,128,129,130]. About 83.9% of bacteria in bee bread honeybee *Apis mellifera* belongs to *Lactobacillus* spp. which is largely composed of *Lb. kunkeei* [128]. However, *Lb. kunkeei* was detected in low quantities in fresh bee bread of solitary bee *Osmia cornuta* and absent in the old bee bread of *Osmia coruta* and bee bread of *H. itama* [15,130].

Recently, Mohammad et al. [15] isolated *Fructobacillus fructosus* from *H. itama* bee bread. *Fructobacillus* spp. is a group of fructophilic LAB, which prefer fructose substrate over glucose [131] and can also be found in *H. itama* honey [132], honeybee *Apis mellifera* bee pollen, and bee bread [133,134] with the capability to utilise plant lignin in vitro [134].

Culture-independent studies help to provide more useful insight into the bee bread bacterial community, taking into account species which are unculturable under controlled condition. Mattila et al. [126] used 454-pyrosequencing technique to sample the active bacteria in the bee bread of honeybee *Apis* without cultural bias. Oenococcus and *Lactobacillus* were the most active microbes in bee bread, comprising 52% and 60% of bacteria, respectively, suggesting its important contribution towards bee pollen conversion.

### 5.2. Bacillus

Lactic acid bacteria have been the integral focus when it comes to bee bread microbial fermentation, but other bacterial species such as *Bacillus* seem to hold its importance in the bee bread metabolic conversion. Although Gilliam [136] found *Bacillus* in honeybee bee bread, the lactic acid production is less efficient than lactic acid bacteria fermentation. The functional role of *Bacillus* in bee bread was not defined until now. One of the early assumptions of *Bacillus* role in bee bread production was to produce enzymes for bee pollen conversion [140]. Few *Bacillus* spp. such as *B. subtilis* [141], *B. megaterium* [142], and *B. licheniformis* [143] have exhibited fermentation ability either using glucose, fructose, or sucrose as substrate. These species have been isolated in bee bread of stingless bee *Melipona fasciata* [140], *H. itama* [15,16], *Tetragonula biroi* Friese [74], honeybee *Apis mellifera* [136], and solitary bee *Osmia cornuta* [130]. However, *Bacillus* fermentation using bee pollen or bee bread is not yet reported.

### 5.3. Yeast

Fermentation is commonly linked with yeast. Yeasts association with fermentation of bee bread has been proposed by researchers. An early study by Gilliam [144] discovered the least yeast count in *Apis mellifera* bee bread as opposed to bee pollen and flower pollen. Several studies had also attempted to describe the yeast communities not only in bee bread but also in bee colonies. In Brazil, yeasts such as *Starmerella* and *Candida* spp. are the most frequently found in the body, pollen, honey, and propolis of stingless bee species of *Tetragonisca angustula, Melipona quadrifasciata*, and *Frieseomelitta varia* [137]. *Starmerella* spp. also has been highly associated with other stingless bee species. Teixeira et al. [137] found *Tetragonisca angustula*′s pollen to harbour *Starmerella meliponinorum*. Meanwhile, Daniel et al. [139] have characterised 25 yeast species with *Starmerella neotropicalis* as the most abundant in *Melipona quinquefasciata* bee bread and bee pollen.

### 5.4. Association of Bee Gut Microbiota and Bee Bread Microbiota

Natural fermentation of bee bread is somehow related to bee gut microbiota [13]. Understanding the bee gut microbiome and the microbial fermenter involved in pollen fermentation could provide valuable information for biotechnological applications [64]. Contribution of bee gut towards bee bread fermentation was first suggested when 6 *Lactobacillus* and 3 *Bifidobacterium* strains were isolated from *A. mellifera* bee bread [129], which are similar to the bacteria isolated from its gut [145].The early assumption was made by theorising bee gut transmission to bee bread through regurgitated honey during bee pollen agglutination process.

The scale of contribution of bee gut towards bee bread microbial inoculation has been studied by Mattila et al. [126]. It was found that only 10% of the bacterial species were commonly shared between bee gut and bee bread. The low impact of bee gut microbiota in contributing a substantial amount of microbes in bee bread fermentation was also supported by Anderson et al. [146] and Corby-Harris et al. [147]. The dominant bacteria in bee bread and honeybee *Apis mellifera* bee gut were also found in fresh flower and nectar, suggesting common horizontal transmission [146]. Nevertheless, it can be concluded that bee gut and also the hive environment contribute towards the bacteria residency in bee bread.

Understanding bee gut microbiome could get complicated because of diverse bacterial communities [145]. By far, the gut microbiota of honeybee *Apis* spp. is widely discussed [148,149] while only a few researchers have attempted to characterise the stingless bee gut microbiota. For instance, the culture-dependent analysis performed on stingless bee *Meliponini beecheii* (Central America), *M. bocandei* (Africa), and *Trigona* sp. (Borneo and Thailand) revealed their honey stomach harbours *Lactobacillus* and *Bifidobacterium* species [150].

The gut microbiome not only varies between species but also between colonies. For example, Leonhardt and Kaltenpoth [151] focused on LAB-bee associated with Australian stingless bee species (*Austroplebeia australis, Tetragonula carbonaria,* and *Tetragonula hockingsi).* Interestingly, the microbiome of these stingless bee is different intercolonies but almost similar to the microbiome of honeybee *Apis mellifera* [150]. Comparison between three different eusocial bees (honeybee, bumblebee, and stingless bee) found *Gilliamella, Bifidobacterium, Lactobacillus* Firm-4 and *Lactobacillus* Firm-5 comprising the core of the gut’s microbiome, with Meliponini as the most diverse one [152].

Bee gut microbiota is maternally inherited [66], acquired through bee faeces or oral trophallaxis [153] and environmental sources such as hive and flower [66]. Functional studies of *A. mellifera* gut microbiota through metagenomic analysis showed a possible link of these microbes to bee protection against pathogens and nutrition acquisition by degrading the pollen wall [12]. Zheng et al. [154] found enrichment of galacturonic acid, a pectin constituent in the honeybee ileum, which suggests pectin degradation. However, no in vitro and in vivo studies have yet to identify the bee gut species responsible for this fermentation process.

Understanding the functional role of bee gut, especially in bee bread fermentation, could be useful. According to Anderson et al. [116], fermentation helps to preserve bee bread environment rather than provide nutrient enrichment. Vásquez et al. [150] demonstrated the ability of LAB strains found in bee bread and honeybee *Apis mellifera* to protect bee bread against bee pathogen *M. plutonius* in vivo and in vitro. However, these studies were heavily based upon honeybee *Apis* sp. More studies should be conducted on stingless bee, whether it will have similar outcomes or not.

## 6. Conclusions

This review paper has summarised the physicochemical properties and health benefits of stingless bee bee bread and has discussed the microbial ecology of bee bread in general in relation to the bee gut. Bee bread is one of the overlooked stingless bee products, highly competing with *Apis* bee pollen in the current market and research focus. The food industry has seen tremendous changes in food trends from processed food to natural products with scientifically proven health benefits. The collective pieces of evidence in recent years have shown the potential of the stingless bee bee bread to be developed as a food ingredient, feed, or a supplement. It is rich in micronutrients, minerals, and phenolic compounds. However, research on their therapeutic values is still scarce. There are wide research opportunities to delve in on the bioactive compounds and their biological properties in vitro and in vivo. More research should be conducted so that an international standard for bee bread of stingless bee could be developed. More research on stingless bee bee bread can add to its commercial value and push to develop the meliponiculture industry. Bee bread has emerged as a reservoir to isolate beneficial microbes that contribute to bee bread preservation and can also provide newfound application in the industries.

## Figures and Tables

**Table 1:** Chemical analysis of bee bread from stingless bee across different geographical locations.

Bee Species	Origin	Botanical Origin	Moisture (%)	Ash (%)	Total Lipid (%)	Protein (%)	Crude Fibres (%)	Total Carbohydrate (%)	Water Activity	pH	References
*Heterotrigona itama*	Malaysia	ND	8.10	1.70	–	47.4	–	55.1	–	–	[77]
*Heterotrigona itama*	Malaysia	ND	–	–	2.17–4.80	17.22–18.37	–	32.74–58.73	–	–	[78]
*Heterotrigona itama*	Malaysia	*Mimosa pudica*, *Sphagneticola trilobata*, *Bidens pilosa*, *Cassia* sp., *Areca catechu*, *Peltophorum pterocarpum*, *Phaleria capitate*, *Cassia siamea*, *Citrus aurantifolia Ageratum conyzoides*	11.09–12.51 ^a^	2.54 ^a^	5.3 ^a^	22.6 ^a^	–	58.2 ^a^	0.73–0.85	–	[22]
*Heterotrigona itama*	Malaysia	ND	30.45	3.28	13.43	30.43	–	22.36	–	–	[79]
*Tetragonula laeviceps*	Thailand	*Trema micrantha*, *Cocos nucifera*	16.1 ^a^	2.30 ^a^	7.4 ^a^	15.5 ^a^	–	58.7 ^a^	-	–	[80]
*Tetragonula testaceitarsis*		*Cocos nucifera*, *Acacia* sp.	31.7 ^a^	2.20 ^a^	5.4 ^a^	17.9 ^a^	–	43.1 ^a^	–	–	
*Lepidotrigona terminata*		*Trema micrantha*, *Cocos nucifera*, *Acacia* sp	25.3 ^a^	1.80 ^a^	5.3 ^a^	14.3 ^a^	–	53.4 ^a^	–	–	
*Lepidotrigona flavibasis*		*Tapirira* sp.	22.8 ^a^	2.20 ^a^	4.9 ^a^	16.7 ^a^	–	53.3 ^a^	–	–	
*Melipona* sp. *aff. eburnea*	Venezuela	Fabaceae Papilionoideae (46%), Malpighiaceae (26%)	48.54	2.33	3.19	18.32	–	27.62	–	–	[81]
*Scaptotrigona* sp. *f. ochrotricha*		Malpighiaceae (54%), Fabaceae Papilionoideae (20%),	43.49	1.94	6.72	16.80	–	31.03	–	–	
*Meliponini seminigra*	Brazil	ND	53.39	4.03 ^a^	10.81 ^a^	37.63 ^a^	9.30 ^a^	25.66 ^a^	0.91	3.70	[82]
*Meliponini interrupta*		ND	37.12	2.74 ^a^	6.47 ^a^	25.00 ^a^	13.65 ^a^	44.27 ^a^	0.85	3.34	
*Tetragonula biroi* Friese	Philippines	ND	14.15–16.73	3.23–3.97 ^a^	2.64–5.43 ^a^	19.55–23.55 ^a^	–	53.05–59.94 ^a^	0.60–0.67	3.73–4.13	[83]
*Tetragonula angustula*	Venezuela	ND	23.56–25.45	1.90–2.22	3.98–5.43	18.10–26.31	–	42.25–49.4	–	–	[84]
*Frieseomelitta* sp. aff. *Varia*	Southern Venezuela	*Antigonon* sp., *Astronium* sp., *Avicennia* sp., *Bidens* sp., *Eupatorium* sp., *Scrophularia nodosa Solanum americanum*	29.96	3.13	3.51	24.72	–	38.68	–	–	[9]
*Melipona compressipes*			32.75	2.85	4.12	21.01	–	39.27	–	–	
*Melipona eburnean*			35.89	2.54	6.03	18.44	–	37.10	–	–	
*Melipona favosa*			29.01	2.92	4.38	22.31	–	41.38	–	–	
*Melipona* sp. *fulva*			31.65	2.45	5.72	19.43	–	40.75	–	–	
*Melipona lateralis kangarumensis*			38.32	2.76	4.80	21.77	–	32.35	–	–	
*Melipona paraensis*			42.74	1.93	5.23	19.08	-	31.02	–	–	
*Melipona mandacaia*	Brazil	ND	36.0	4.9	–	21.0	3.6	–	0.86	3.49	[85]
*Scaptotrigona mexicana*	Cañada Blanca, Mexico	*Heliocarpus* sp., *Bursera simaruba, Chamaecrista* sp., *Desmodium adscendens, Desmodium tortuosum*, *Eugenia capuli*, *Bidens pilosa*, *Vernonia* sp., *Pouteria* sp., *Verbesina* sp., *Coccoloba* sp.	24.6	3.1	0.46	22.01	–	31.99	–	3.46	[86]
*Scaptotrigona mexicana*	Manuel León, Mexico	*Bursera simaruba*, *Parthenium fruticosum*, *Helianthus* sp., *Spondias mombin*, *Solanum* sp., *Chamaecrista* sp., *Serjania* sp., *Heliocarpus* sp., *Vernonia* sp., *Pithecellobium* sp. *Coccoloba* sp., *Bidens pilosa.*	15.5	2.5	1.1	20.49	-	33.10	–	3.61	[86]
*Scaptotrigona mexicana*	Fortín de las Flores, Mexico	*Spondias mombin*, *Dendropanax arboreus*, *Solanum* sp., *Verbesina* sp., *Heliocarpus* sp., *Pouteria* sp., *Cordia* sp., *Vernonia* sp., *Eugenia* sp., *Dysphania ambrosioides*,	26.7	2.9	1.1	21.06	–	35.02	–	3.64	[86]
*Melipona scutellaris*	Brazil	*Tapirira guianensis*, *Spondias mombin*, *Syagrus* sp., *Hylocereus undatus*, *Pueraria phaseoloides*, *Mimosa caesalpiniifolia*, *Pithecellobium dulce*, *Ossaea* sp., *Miconia* sp., *Psidium* sp., *Corymbia torrelliana*, *Myrcia obovate*, *Syzygium samarangense*, *Campomanesia* sp., *Eugenia stipitate*, *Eugenia uniflora*, *Plinia cauliflora*, *Solanum macrocarpon*, *Solanum stipulaceum*, *Solanum* sp., *Cestrum* sp., *Allophylus* sp.	44.71	1.84	4.25	23.88	0.87	24.48	0.92	3.75	[71]
*Melipona scutellaris*	Brazil	*Cocos nucifera*, *Spondias*, *Tapirira*, *Bidens*, *Vernonia*, *Protium*, *Terminalia*, *Erythroxylum*, *Acalypha*, *Croton*, *Acacia*, *Caesalpinia*, *Centrosema*, *Chamaecrista*, *Desmodium*, *Leucaena*, *Macroptilium*, *Mimosa caesalpiniifolia*, *Mimosa pudica*, *Mimosa quadrivalvis*, *Mimosa tenuiflora*.	47.3–55.7	3.45–5.90	2.43–7.94	10.19–24.02	–	10.85–28.89	–	3.28–3.99	[33]

^a^ Dry weight; ND = not determined.

**Table 2:** Essential amino acid in stingless bee bee bread (g/100 g).

Bee Species	Country	Instrument	Phe	Val	His	Met	Iso	Leu	Thr	Ala	Try	References
*Heterotrigona itama*	Malaysia ^1^	HPLC	1.84–2.80	1.00–1.18	0.78–1.15	0.37–0.53	0.76–0.83	1.61–1.83	1.52–2.00	1.00–1.06	ND	[22]
*Tetragonula laeviceps*	Philippines ^1^	MS-GC	1.63	0.65	0.96	0.12	0.88	1.62	0.17	0.40	0.96	[80]
*Melipona subnitida*	Brazil ^2^	HPLC-PDA	0.36–0.41	0.29–0.41	0.70–0.73	0.14–0.17	0.14–0.21	0.61	0.23–0.30	0.91–0.93	0.70–0.73	[87]

Phe: phenylalanine, Val: valine, His: histidine, Met: methionine, Iso: isoleucine, Leu: leucine, Thr: threonine, Ala: alanine, Try: tryptophan; HPLC: high-performance liquid chromatography, PDA: photodiode-array detector, MS: mass spectrometry, GC: gas chromatography; ND = not determined; ^1^ For botanical origin, refer to Table 1; ^2^ Botanical origin not determined.

**Table 3:** Sugar content in stingless bee bee bread (g/100 g).

Bee Species	Country	Botanical Origin	Instrument	Glucose	Fructose	Sucrose	Maltose	Mannitol	References
*Heterotrigona itama*	Malaysia	*Mimosa pudica*, *Sphagneticola trilobata*, *Bidens pilosa*, *Cassia* sp., *Areca catechu*, *Peltophorum pterocarpum*, *Phaleria capitate*, *Cassia siamea*, *Citrus aurantifolia Ageratum conyzoides*	HPLC-ELSD	10.27–12.40	0.40–1.49	0.60–2.10	0.70–2.00	–	[22]
*Melipona subnitida*	Brazil	ND	HPLC-RID	–	–	–	–	20.80–31.00	[87]
*Melipona subnitida*	Brazil	*Mimosa gemmulata*, *Mimosa verrucada*, Fabaceae, Scrophulariaceae (species not identified)	Column chromatography-TLC-UV	–	–	–	–	34.90	[101]
*Tetragonula biroi*	Philippines	ND	HPAEC-PAD	0.25–1.89	4.39–9.58	4.29–26.04	0.82–2.04	5.57–20.85	[83]

HPLC: high-performance liquid chromatography, ELSD: evaporative light scattering detector, RID: refractive index detector, TLC: thin-layer chromatography, HPAEC: high-performance anion exchange chromatography, PAD: pulsed amperometric detector.

**Table 4:** Bioactive compounds in stingless bee bee bread.

Bee Species	Origin	Botanical Origin	Instrument	Compound	References
*Melipona subnitida*	Brazil	*Mimosa gemmulata*, *Mimosa verrucada*, Fabaceae, Scrophulariaceae (species not identified)	Column chromatography	6-naringenin, 7-isorhamnetin, 2-tricetin, 3-selagin, 4-8-methoxyherbacetin	[101]
*Heterotrigona itama*	Malaysia	ND	LC-MS	Caffeic acid, ferulic acid, kaempferol, apigenin, and isorhamnetin	[104]
*Frieseomelitta* sp. aff. Varia,	Southern Venezuela	*Antigonon* sp., *Astronium* sp., *Avicennia* sp., *Bidens* sp., *Eupatorium* sp., *Scrophularia nodosa Solanum americanum*	HPLC-UV	Kaempferol, luteolin, quercetin	[9]
*Melipona compressipes,*				Kaempferol, luteolin, 8 methoxykaempferol	
*Melipona eburnean*				Genkwanin, 8 methoxykaempferol	
*Melipona favosa,*				Quercetin	
*Melipona* sp. *fulva*				Kaempferol, 8 methoxykaempferol, quercetin	
*Melipona lateralis kangarumensis*				Kaempferol, luteolin, 8 methoxykaempferol, quercetin	
*Melipona paraensis*				Genkwanin, luteolin	
*Melipona rufiventris*	Brazil	ND	Column chromatography—NMR	*p*-hydroxycinnamic acid, dihydroquercetin, isorhamnetin, isorhamnetin-3-*O*-(6”-*O*-E-*p*-coumaroyl)-β-*D*-glucopyranoside, luteolin, and quercetin	[29]
*Scaptotrigona affinis postica*	Brazil	ND	LC-ESI-IT-MS/MS	Gluconic acid, digalloylshikimic acid, quercetin-3,4-diglucoside, apigenin-6-C-glucoside, isoorientin-2”-*O*-rhamnoside, kaempferol 3,7-di-*O*-rhamnoside, ellagic acid, monogalloylglucose, protocatechuic acid 3-glucoside, procyanidin dimmer digallate (a-type)	[105]
*Melipona fasciculata*	Brazil	ND	LC-ESI-IT-MS/MS	Gluconic acid derivate, gluconic acid, kaempeferol derivative, kaempferol, 6-hydroxykaempferol 3,6-diglucoside 7-glucuronide, ellagic acid dimer, quercetin 3,4′-diglucoside, ellagic acid, protocatechuic acid 3-glucoside, linolenic acid, linoleic acid	[106]
*Tetragonula biroi* Friese	Philippines	ND	HPLC-DAD	Rutin, quercetin-3-*O*-β-*D*-glucoside, and quercetin	[107]

LC: liquid chromatography, MS: mass spectrometry, HPLC: high-performance liquid chromatography, UV: ultraviolet, NMR: nuclear magnetic resonance, ESI: electrospray ionisation, IT: ion trap, DAD: photodiode array detection.

**Table 5:** Mineral composition in stingless bee bee bread.

Bee Species	Country	Instrument	K	P	Na	Mg	Ca	Mn	Fe	Cu	Zn	Se	References
*Heterotrigona itama*	Malaysia	ICP-MS	6524.9	6402.28	139.70	1635.4	1547.31	61.66	126.43	12.7671	60.62	0.26	[22]
*Tetragonula laeviceps*	Brazil	ICP-OES	5656.0	–	89.9	1160.0	2566.0	–	–	–	–	–	[80]
*Tetragonula testaceitarsis*			4594.7	–	133.5	1318.0	2904.0	–	–	–	–	–	
*Lepidotrigona terminata*			4606.3	–	77.2	1176.0	2507.3	–	–	–	–	–	
*Lepidotrigona flavibasis*			5125.7	–	81.7	1315.3	2719.7	–	–	–	–	–	
*Melipona subnitida*	Brazil	FAAS	5918.5–13,366.6	–	–	975.4–2166.1	1864.1–3424.9	35.1–75.0	16.4–33.5	0.8–1.9	36.4–71.2	–	[87]
*Scaptotrigona mexicana*	Mexico	ICP-OES	2222.5–2836.9	2736.1–3657.3	–	–	–	–	–	–	–	–	[86]
*Tetragonula biroi*	Philippines	ICP-MS	–	–	–	1763.58–2407.65	3780.28–5186.89	42.58–266.90	70.37–123.77	9.52–12.02	45.16–56.25	–	[83]

K: potassium, P: phosphorus, Na: sodium, Mg: magnesium, Ca: calcium, Mn: manganese, Fe: iron, Cu: copper, Zn: zinc, Se: selenium; ICP: inductively coupled plasma, MS: mass spectrometry, OES: optical emission spectrometry, FAAS: flame atomic absorption spectrometry.

**Table 6:** Health benefits of stingless bee bee bread.

**Stingless Bee Species**	**Origin**	**Sample Preparation**	**Method**	**Explanation**	**Reference**
**Antimicrobial Activity**					
*Melipona compressipes manaosensis*	Maues, Amazonas, Brazil	Bee bread from 3 sampling sites was extracted using hexane (Hex), EtOH, and MetOH	Agar well diffusion and MIC	All sample inhibited *Mycobacterium smegmatis* (ATCC 607) and *Pseudomonas aeruginosa* (ATCC 27853). Only bee bread hex, EtOH, and MetOH extract from Maues 3 inhibited *Streptococcus oralis* (ATCC 10557). *C. albicans* was sensitive to all extracts. Most of the extracts tested showed MIC greater than 1000 µg/mL. Bee bread EtOH Maues 1 and MetOH Maues 3 showed larvicidal activity *C. quinquefasciatus* LC_50_ 218.2 and 228.8 µg/mL.	[112]
*Heterotrigona itama*	Malaysia	Bee bread was extracted in hexane and 70% EtOH	Disc diffusion method	Gram-positive bacteria (*B. cereus* and *S. aureus*) showed bigger inhibition zone than Gram-negative bacteria (*E. coli* and *Salmonella*)	[113]
*Austroplebeia australis, Tetragonula carbonaria, Tetragonula hockingsi*	Australia	Extraction in MetOH and EtOH	Agar well diffusion and MIC	All extracts inhibited *Staphylococcus aureus* (ATCC 25923), *Bacillus subtilis* (ATCC 11778), *Enterobacter cloacae* (ATCC 13047), *Escherichia coli* (ATCC 25922), and *Pseudomonas aeruginosa* (ATCC 27853) except for *A. australis* MetOH against *P. aeruginosa*, *T. hockingsi* MetOH against *E. coli*, and *T. hockingsi* ethanolic extract against *E. coli*. MIC values of ethanolic extracts were lower than methanolic extracts of pot-pollen	[100]
*Frieseomelitta* sp. aff. *Varia*, *Melipona compressipes*, *Melipona eburnean*, *Melipona favosa*, *Melipona* sp. *fulva*, *Melipona lateralis kangarumensis*, *Melipona paraensis*	Australia	Extraction in 95% EtOH	Agar well diffusion	All extracts inhibited *Bacillus subtilis* (ATCC 11778), *Staphylococcus aureus* (ATCC 25923), *Escherichia coli* (ATCC 25922), *Enterobacter cloacae* (ATCC 13047), and *Pseudomonas aeruginosa* (ATCC 27853).	[114]
**Antioxidant Activity**					
*Heterotrigona itama*	Malaysia	Extraction in 95% EtOH	DPPH	IC_50_: 300 µg/mL	[79]
*Trigona apicalis*, *Trigona itama*, *Trigona thoracica*	Malaysia	Extraction in MetOH	DPPH	IC_50_: *Trigona apicalis* 1.05 ± 0.01 mg/mL, *Trigona itama* 3.24 ± 0.03 mg/mL, *Trigona thoracica* 0.86 ± 0.01 mg/mL	[99]
*Heterotrigona itama*	Malaysia	Bee bread was extracted in hexane and 70% EtOH at concentration 150 µL/mL	DPPH and ABTS	DPPH assay: EtOH bee bread was 93.60 ± 0.0% inhibition and Hex bee bread was 83.81 ± 0.05% inhibition. ABTS assay: EtOH bee bread showed 97.95% inhibition and Hex bee bread was 71.23%.	[113]
*Melipona asilvai* Moure, *M. quadrifasciata anthidioides* Lepeletier, *M. scutellaris* Latreille, *M. subnitida* Ducke, *Frieseomelitta varia* Lepeletier, *Tetragona clavipes* Fabricius and *Plebeia* sp.	Brazil	Extraction in 70% EtOH at concentration 50 mg/mL	DPPH, FRAP and inhibition of β-carotene	FRAP assay: *T. clavipes* recorded the highest with 123.4 mgGAE/100 g. Inhibition of β-carotene: *M. q. anthidioides* (83.3%) was the highest followed by *M*. *scutellaris* (76.5%). DPPH values were not stated.	[47]
*Frieseomelitta* sp. aff. *Varia*, *Melipona compressipes*, *Melipona eburnean*, *Melipona favosa*, *Melipona* sp. *fulva*, *Melipona lateralis kangarumensis*, *Melipona paraensis*	Brazil	Extraction in 95% EtOH	ABTS, Fenton type reaction, and hydroxyl radical	Fenton-type reaction: 0.91–1.25 mM uric acid equivalents/100 g pot-pollen. ABTS assays: 193.2 and 771.0 μmoles Trolox equivalents/100 g bee bread. Hydroxyl radical inhibition: 54.08% and 97.32% inhibition/100 g bee bread. The highest antioxidant activity was *Melipona lateralis kangarumensis* for all three tests.	[9]
*Tetragonisca angustula*	Venezuela	Extraction in 95% EtOH	ABTS, Fenton type reaction, and hydroxyl radical	Fenton-type reaction: 0.74 and 1.12 mM uric acid equivalent/100 g bee bread. ABTS assays: values were between 401.8 and 500.4 μmoles Trolox equivalents/100 g bee bread. Hydroxyl radical inhibition: 30.0–60.1% inhibition/100 g bee bread. High positive correlations (0.802–0.921) between polyphenol contents and antioxidant activity measured by the three methods.	[84]
*Austroplebeia australis*, *Tetragonula carbonaria*, *Tetragonula hockingsi*	Australia	Bee bread was extracted in MetOH and EtOH	ABTS, Fenton type reaction, and hydroxyl radical	The hydroxyl radical inhibition: 48.6–71.3%/100 g bee bread Fenton-type reaction: 1.00 and 1.05 mM uric acid/100 g bee bread ABTS assay: 229.6 and 426.4 μmol TEAC/100 g of bee bread Overall, EtOH showed higher antioxidant activity than MetOH, but the results were not statistically different. A positive correlation was found between the antioxidant activity measured with three methods and the polyphenol content of bee bread.	[100]
*Melipona fasciculata*	Brazil	Extraction in 70% EtOH	DPPH, FRAP, and ABTS	DPPH: IC_50_ 117–597.73 µg/mL. FRAP: 0.15–0.99 mmol Fe^2+/^g ABTS: IC_50_ 34.40–235.19 µg/mL	[106]
*Scaptotrigona affinis postica*	Brazil	Extraction in 70% EtOH	DPPH, FRAP, and ABTS	DPPH: IC_50_ 273.08 ± 1.43 µg/mL. FRAP: 0.71 ± 0.04 Fe^2+/^g ABTS: IC_50_ 87.29 ± 0.06 µg/mL	[105]
*Tetragonula biroi* Friese	Thailand	Extraction in MetOH using two different methods (maceration and sonication)	DPPH, FRAP, ABTS, LPO	DPPH: EC_50_ 3.62–43.04 mg pollen/mL (macerated) and 3.32 to 44.50 mg pollen/mL (sonicated) FRAP: 97.13–251.17 µmol Fe^2^+ (macerated) and 95.51–256.19 µmol Fe^2^+ (sonicated). ABTS: 8.90–62.06 µmol TE/g pollen (macerated) and 8.08–62.24 µmol TE/g pollen (sonicated). LPO: 33.97–44.72% (macerated) and 37.09–47.34% (sonicated).	[107]
**Others Therapeutic Properties**					
***Health Benefits***	**Animal Models**	**Research Design**	**Treatment**	**Effect**	
Antidiabetic activity	Thirty-six male Sprague Dawley rats	Study the antiatherogenic effect of bee bread in HFD-induced obese rats. Bee bread of *Heterotrigona itama* was administered via oral gavage.	G1: Normal G2: HFD G3: HFD + bee bread (high-fat diet and 0.5 g/kg/day bee bread) G4: HFD + orlistat (high-fat diet and 10 mg/kg/day orlistat) groups	Bee bread (0.5 g/kg) prevented or suppressed HFD-induced obesity in a manner that was similar or even better than orlistat (at 10 mg/kg). Bee bread treatment; ↓ Lee obesity index, ↓ TC, ↓ LDL, ↓ FAS, ↓ atherogenic index, ↓ oxLDL, ↓ MDA and significantly increased aortic antioxidant enzymes activities (SOD and GPx) in high-fat diet (HFD)-induced obese rats. En face aorta images showed smaller adipocytes sizes, and absence of atherosclerotic plaque in obese rats supplemented with bee bread.	[104]
Antidiabetic activity	Thirty-six male Sprague Dawley rats	Study the bee bread effect on serum renal function parameters, oxidative stress, inflammatory, and Bax in the kidneys of HFD obese rats. Bee bread of *Heterotrigona itama* was administered via oral gavage.	G1: Control: received rat diet and water (1 mL/kg); G2: HFD group: received HFD and water (1 mL/kg) G3 and G4: bee bread preventive or orlistat preventive G5 and G6: received HFD and BB (0.5 g/kg) or HFD and orlistat (10 mg/kg); BB or orlistat treatment: received BB (0.5 g/kg) or orlistat (10 mg/kg)	Bee bread (0.5 g/kg) has shown to ↓ body weight, ↓ BMI, ↓ Lee obesity index, ↓ MDA, ↑ TAA, ↑ SOD, ↑GPx, ↑ GST. ↓ expression of NFkB, TNF-a, IL-1b, and Bax gene.	[118]
Anti-inflammatory activity	Adult male Mus musculus mice with dextran-induced and carrageenan-induced paw oedema	Study the bee bread effect on induced-oedema paw mice model. Bee bread EetOH extract of *Melipona fasciculata* was administered orally and was monitored for 5 h hourly.	G1: saline (10 mL/kg) G2: bee bread extract 250 mg/kg G3: bee bread extract 500 mg/kg) G4: cyproheptadine for dextran-induced treatment (10 mg/kg) and indomethacin for carrageenan-induced (10 mg/kg)	Carrageenan-induced paw oedema test: G3 significantly ↓ mice paw oedema by 52% compared to control in 1 h. After 5 h, G2, G3, and G4 reduced oedema by better (*p* < 0.001) than G1. Dextran-induced paw oedema test: G3 significantly ↓ mice paw oedema by 84% compared to G4 in 1 h. After 5 h, G2, G3, and G4 reduced oedema better (*p* < 0.001) than G1.	[106]
Anti-inflammatory activity	Adult male Mus musculus mice with dextran-induced and 1% carrageenan-induced paw oedema	Study bee bread effect on induced-oedema paw mice model. Bee bread EtOH extract of *Scaptotrigona affinis postica* was administered orally and was monitored for 5 h with 1 h interval	G1: saline (10 mL/kg) G2: bee bread extract (250 mg/kg) G3: bee bread extract (500 mg/kg) G4: cyproheptadine for dextran-induced treatment (10 mg/kg) and indomethacin for carrageenan-induced (10 mg/kg)	Carrageenan-induced paw oedema test: After 5 h, G2, and G3 significantly ↓ mice paw oedema by similar to G5, but better than G1. Dextran-induced paw oedema test: After 5 h, G2, and G3 significantly ↓ mice paw oedema by similar to G5 but better than G1.	[105]
Antinociceptive activity	Adult male Mus musculus mice injected with 0.8% acetic acid and 2.5% formalin separately	Bee bread EtOH extract of *Melipona fasciculata* was administered orally.	G1: saline (10 mL/kg) G2: bee bread extract (250 mg/kg) G3: bee bread extract (500 mg/kg) G4: indomethacin (10 mg/kg)	Acetic acid writhing test: G3 was more efficient than G4 to reduce abdominal contortions produced, with less than 58% writhing (*p* < 0.05). Formalin test: In neurogenic phase, G2 and G3 ↓ time that the animals passed licking/biting the induced paw in 42% and 47%, better than G1 and G4. In inflammatory phase, G2, G3, and G4 showed no difference (*p* > 0.05) to the mice licking/biting time.	[106]
Antinociceptive activity	Adult male Mus musculus mice injected with 0.8% acetic acid and 2.5% formalin separately	Bee bread EtOH extract of *Scaptotrigona affinis postica* was administered orally.	G1: saline (10 mL/kg) G2: bee bread extract (250 mg/kg) G3: indomethacin (10 mg/kg)	Acetic acid writhing test: G2 ↓ the number of abdominal writhing’s by 52% compared to G1 (*p* < 0.05), but equal to G3. Formalin test: In neurogenic phase, G2 ↓ time that the animals passed licking/biting the induced paw by 57%, better than G1 and 51% compared to G3. In inflammatory phase, G2 ↓ licking/biting responses by 99%, better than G1 and 96% compared to G3.	[105]

EtOH: ethanol, MetOH: methanol, MIC: minimum inhibitory concentration, DPPH: 1,1-diphenyl-2-picrylhydrazyl radical (DPPH), FRAP: ferric reducing-antioxidant power, LPO: lipid peroxidation, TEAC: Trolox equivalent antioxidant capacity, HFD: high-fed diet, TC: total cholesterol, LDL: low-density lipoprotein, FAS: fatty acid synthase, oxLDL: oxidised-LDL, MDA: malondialdehyde, SOD: superoxide dismutase, GPx: glutathione peroxidase, BMI: body mass index, TAA: total antioxidant activity, GST: glutathione-S-transferase, NFkB: nuclear factor kappa B, TNF-a: tumour necrosis factor alpha, IL-1b: interleukin-1-b, and Bax: B-cell associated protein.

**Table 7:** Lactic acid bacteria, nonlactic acid bacteria, and yeasts associated with stingless bee bee bread.

Bacterium	Bee Species	References
**Lactic Acid Bacteria (LAB)**		
*Lactobacillus* sp.	*Apis mellifera* ^1^	[128,129,134]
	*Apis* sp. ^1^	[123,126]
*Lactobacillus kunkeei*	*Apis mellifera* ^1^	[14,128,135]
	*Osmia cornuta* ^3^	[130]
	*Apis* sp. ^1^	[123]
*Lactobacillus mucosae*	*Apis mellifera* ^1^	[128]
*Lactobacillus musae*	*Heterotrigona itama* ^2^	[15]
*Lactobacillus crustorum*	*Heterotrigona itama* ^2^	[15]
*Lactobacillus mindensis*	*Heterotrigona itama* ^2^	[15]
*Lactobacillus casei*	*Apis mellifera* ^1^	[127]
*Leuconostoc mesenteroides*	*Heterotrigona itama* ^2^	[15]
*Enterococcus durans*	*Osmia cornuta* ^3^	[130]
*Enterococcus faecalis*	*Osmia cornuta* ^3^	[130]
	*Heterotrigona itama* ^2^	[15]
*Enterococcus faecium*	*Osmia cornuta* ^3^	[130]
*Fructobacillus* sp.	*Apis mellifera* ^1^	[134]
*Fructobacillus fructosus*	*Heterotrigona itama* ^2^	[15]
*Weisella* sp	*Osmia cornuta* ^3^	[130]
*Weisella confusa*	*Osmia cornuta* ^3^	[130]
*Weisella viridescens*	*Osmia cornuta* ^3^	[130]
*Oenococcus*	*Apis* sp. ^1^	[126]
**Non-LAB**		
*Bifidobacterium* sp.	*Apis mellifera* ^1^	[14]
	*Apis* sp. ^1^	[126]
*Bifidobacterium asteroides*	*Apis mellifera* ^1^	[135]
*Bacillus* sp.	*Osmia cornuta* ^3^	[130]
*Bacillus megaterium*	*Tetragonula biroi Friese* ^2^	[74]
*Bacillus thuringiensis*	*Osmia cornuta* ^3^	[130]
*Bacillus licheniformis*	*Osmia cornuta* ^3^	[130]
	*Apis mellifera* ^1^	[136]
*Bacillus pumilus*	*Osmia cornuta* ^3^	[130]
	*Apis mellifera* ^1^	[136]
	*Tetragonula biroi Friese* ^2^	[74]
	*Heterotrigona itama* ^2^	[15]
*Bacillus flexus*	*Tetragonula biroi Friese* ^2^	[74]
*Bacillus coagulans*	*Tetragonula biroi Friese* ^2^	[74]
*Bacillus subtilis*	*Osmia cornuta* ^3^	[130]
	*Tetragonula biroi Friese* ^2^	[74]
	*Apis mellifera* ^1^	[136]
*Bacillus safensis*	*Heterotrigona itama* ^2^	[15]
*Bacillus amyloliquefaciens*	*Heterotrigona itama* ^2^	[15]
	*Tetragonula biroi Friese* ^2^	[74]
*Bacillus cereus*	*Heterotrigona itama* ^2^	[15]
	*Tetragonula biroi Friese* ^2^	[74]
**Yeast**		
*Starmerella meliponinorum*	*Tetragonisca angustula* ^2^	[137,138]
*Starmerella neotropicalis*	*Melipona quinquefasciata* ^2^	[139]

^1^ Honeybee, ^2^ Stingless bee, ^3^ Solitary bee.
